# Genomic characterization of sporadic isolates of the dominant clone of *Mycobacterium abscessus* subspecies *massiliense*

**DOI:** 10.1038/s41598-021-94789-y

**Published:** 2021-07-28

**Authors:** Rebecca M. Davidson, Jeanne B. Benoit, Sara M. Kammlade, Nabeeh A. Hasan, L. Elaine Epperson, Terry Smith, Sruthi Vasireddy, Barbara A. Brown-Elliott, Jerry A. Nick, Kenneth N. Olivier, Adrian M. Zelazny, Charles L. Daley, Michael Strong, Richard J. Wallace

**Affiliations:** 1 Center for Genes, Environment and Health, National Jewish Health, 1400 Jackson St., Denver, CO 80206 USA; 2 Department of Microbiology, University of Texas Health Science Center at Tyler, Tyler, TX USA; 3 Department of Medicine, National Jewish Health, Denver, CO USA; 4 Pulmonary Branch, National Heart, Lung, and Blood Institute, National Institutes of Health, Bethesda, MD USA; 5 Department of Laboratory Medicine, Clinical Center, National Institutes of Health, Bethesda, MD USA

**Keywords:** Computational biology and bioinformatics, Evolution, Genetics, Microbiology, Diseases

## Abstract

Recent studies have characterized a dominant clone (Clone 1) of *Mycobacterium abscessus* subspecies *massiliense* (*M. massiliense*) associated with high prevalence in cystic fibrosis (CF) patients, pulmonary outbreaks in the United States (US) and United Kingdom (UK), and a Brazilian epidemic of skin infections. The prevalence of Clone 1 in non-CF patients in the US and the relationship of sporadic US isolates to outbreak clones are not known. We surveyed a reference US Mycobacteria Laboratory and a US biorepository of CF-associated Mycobacteria isolates for Clone 1. We then compared genomic variation and antimicrobial resistance (AMR) mutations between sporadic non-CF, CF, and outbreak Clone 1 isolates. Among reference lab samples, 57/147 (39%) of patients with *M. massiliense* had Clone 1, including pulmonary and extrapulmonary infections, compared to 11/64 (17%) in the CF isolate biorepository. Core and pan genome analyses revealed that outbreak isolates had similar numbers of single nucleotide polymorphisms (SNPs) and accessory genes as sporadic US Clone 1 isolates. However, pulmonary outbreak isolates were more likely to have AMR mutations compared to sporadic isolates. Clone 1 isolates are present among non-CF and CF patients across the US, but additional studies will be needed to resolve potential routes of transmission and spread.

## Introduction

*Mycobacterium abscessus* is currently divided into three subspecies including subsp. *abscessus*, subsp. *massiliense* and subsp. *bolletii*^1^. Closely related strains of *Mycobacterium abscessus* subspecies *massiliense* (hereafter *M. massiliense*) were identified from three widely separated outbreaks by comparative genomics^2^. The outbreaks included cystic fibrosis (CF) related pulmonary isolates from a hospital in the United Kingdom (UK)^3^, CF pulmonary isolates from a United States (US) clinic^4,5^, and isolates associated with an epidemic of soft tissue infections in Brazil^6,7^. Very few non-outbreak related strains were identified in the study. The authors recommended screening all CF isolates for relatedness to these outbreak strains and suggested a multi locus sequence typing (MLST) method including two single nucleotide polymorphisms (SNPs) in the *rpoB* gene and a SNP in the *secA1* gene.

A subsequent global population study using whole genome sequencing (WGS) revealed that the genetic subtype described among the widely separated outbreaks^2^ (hereafter called Clone 1) was the most prevalent *M. massiliense* genotype, or dominant circulating clone, among CF patients across several countries in Europe, Australia and one site in the US^8^. Dominant clones were found to have higher proportions of antimicrobial resistance (AMR) mutations to amikacin and clarithromycin compared to diverse, unclustered isolates. In contrast, a population genomics study by the Colorado Research and Development Program (CO-RDP) of *M. massiliense* isolates from US CF centers across 14 states showed that Clone 1 was not the most prevalent genotype (in only 17% of subjects) and that AMR mutations were relatively rare^9^. A genomic analysis of 188 *M. massiliense* isolates from soft tissue infections across 10 Brazilian states confirmed that the Brazilian strains are closely related to Clone 1 strains from the UK pulmonary outbreak^10^. However, they appeared to be a unique lineage with smaller genomes than other *M. massiliense* and contained distinct plasmids called pMAB01^11^ and pMAB02^10^. It is currently unknown if Clone 1 is present among non-CF pulmonary isolates or extrapulmonary infections outside of Brazil.

For this study, consecutive non-CF and CF clinical isolates of *M. abscessus,* including subsp. *abscessus*, subsp. *massiliense* and subsp. *bolletii,* were identified over a 3-year period at the Mycobacteria/Nocardia Research Laboratory at the University of Texas Health Science Center at Tyler (UTHSCT), a national reference laboratory for nontuberculous mycobacteria (NTM). Isolates of *M. massiliense* were screened by MLST for sequence characteristics of the Clone 1 genotype, and selected isolates were analyzed by WGS. Non-CF and CF *M. massiliense* isolates with the MLST profile from a second US site, the National Institutes of Health (NIH) Clinical Center, were also analyzed by WGS. Isolates sequenced for this study (n = 34) were analyzed along with previously sequenced US CF isolates from the CO-RDP (n = 41) and isolates from 11 published studies (n = 25) to compare sporadic US isolates from various clinical settings to known outbreak isolates. Our objectives were to (1) test whether Clone 1 isolates exist in non-CF populations in the US and (2) compare the prevalence of Clone 1 between the UTHSCT and CO-RDP isolate cohorts. Moreover, we hypothesize that sporadic US Clone 1 isolates will differ from previously identified outbreak isolates of *M. massiliense* in terms of genomic similarity and antimicrobial resistance.

## Materials and methods

### Study population

For the UTHSCT samples, the study protocol was submitted to the human subjects committee at the UTHSCT and was deemed exempt from patient informed consent given the de-identification of all isolates in the study. For NIH samples, patients were enrolled in Institutional Review Board-approved protocols at the National Institutes of Health. For CO-RDP samples, the study was reviewed and approved by the National Jewish Health (NJH) Institutional Review Board (HB-0063). All experiments were performed in accordance with relevant guidelines and regulations.

### Species identification and subtyping of *M. massiliense* isolates at the UTHSCT

The Mycobacteria/Nocardia Research Laboratory at the UTHSCT is a national reference laboratory for adult and pediatric isolates of NTM. Consecutive isolates submitted between November 2014 and August 2017 for identification to species were screened for isolates of *M. massiliense.* Isolates of *M. abscessus* (including subspecies *M. massiliense*) were first identified to species and subspecies levels by *rpoB* partial gene sequencing^12^ and/or *erm*(41) gene sequencing^13,14^. Isolates of *M. massiliense* were further screened as Clone 1 using the SNP signature described previously including the two base pair (bp) substitution of the region 5 *rpoB* gene sequence of the type strain of *M. abscessus* ATCC 19977^T2^. MLST was performed by sequencing the region 5 *rpoB* gene^12^, the *erm*(41) gene^14^, and the *secA*1 gene^15^. A subset of 30 isolates from 25 patients was sent to NJH for WGS including 23 isolates from 23 patients (using the first isolate collected in the 2014–2017 study period). Also included among WGS samples were two longitudinal isolates from one patient, and a set of five longitudinal isolates from one patient. Inclusion criteria for WGS were two SNPs in the *rpoB* gene (2569 C/T and 2670 T/C) and one SNP in the *secA1* gene (821 G/T).

### Patients at the UTHSCT

Details of the patients and their isolates were obtained from information provided with laboratory submission. These included age, sex, presence or absence of a diagnosis of CF, underlying disease including nodular bronchiectasis, specimen source, geographical location, and date of isolation. A checkbox for the presence or absence of CF was on the sheet, although the details of their CFTR mutations were not requested.

### Subtyping of *M. massiliense* isolates from the NIH mycobacteriology laboratory

Strains were identified as *M. massiliense* and further subtyped by partial sequencing of *rpoB*, *hsp65* and *secA1* genes^16^ and a *M. abscessus* subspecies PCR identification scheme^17^. A subset of MLST-identified Clone 1 and non-Clone 1 isolates (n = 4) was sent to NJH for WGS. Two were pulmonary isolates from persons with CF. The other two were pulmonary and extrapulmonary isolates from non-CF patients.

### Whole genome sequencing

Genomic DNA was isolated from bacterial pellets using a method described previously^18^. Sequencing libraries were prepared with 5 ng of genomic DNA and the Nextera XT Library Prep Kit (Illumina Inc., San Diego CA). Libraries were sequenced on the Illumina MiSeq using 2 × 300 bp paired-end sequencing chemistry. A total of 34 sporadic *M. massiliense* isolates were sequenced in this study, including 30 isolates from 25 patients at UTHSCT, and 4 isolates from 4 patients at the NIH (Table 1). The sequencing cohort includes pulmonary isolates from patients with and without a CF diagnosis, and extrapulmonary isolates cultured from blood, ear and wounds. The patients resided in 11 states across the US and are not associated with any known outbreaks. Thus, these cases are considered to be sporadic (i.e. epidemiologically unrelated).

**Table 1:** *M. massiliense* isolate cohort for genomic analyses.

Mycobacteria laboratory	Source, disease	No. isolates (No. patients)	References
**Sporadic Clone 1 isolates**			
UTHSCT	Pulmonary, CF	12 (8)	This study
UTHSCT	Pulmonary, non-CF	9 (9)	This study
UTHSCT	Extrapulmonary	4 (4)	This study
NIH	Pulmonary, non-CF	1	This study
NIH	Extrapulmonary	1	This study
**Published Clone 1 isolates**			
Papworth, UK, outbreak	Pulmonary, CF	8 (5)	^3^
Seattle, WA, outbreak	Pulmonary, CF	3 (3)	^2^
Global study (UK, Australia, US)	Pulmonary, CF	6 (6)	^8^
Great Ormand Hospital, UK	Pulmonary, CF	1	^19^
Birmingham, UK	Pulmonary, CF	1	^20^
CO-RDP	Pulmonary, CF	12 (9)	^9,21^
Rio de Janeiro, Brazil, epidemic	Extrapulmonary	1	^22^
Para, Brazil, epidemic	Extrapulmonary	1	^10^
**Non-Clone 1 control isolates**			
UTHSCT	Pulmonary, CF	3 (2)	This study
UTHSCT	Pulmonary, non-CF	2 (2)	This study
NIH	Pulmonary, CF	2 (2)	This study
CO-RDP	Pulmonary, CF	29 (25)	^9,21^
5S-0304	Unknown	1	Unpublished
Marseille, France—CCUG48898^T^	Pulmonary, non-CF	1	^23^
University Malaya, Malaysia—M154	Pulmonary	1	^24^
Seoul University, Korea—ASAN50594	Pulmonary	1	^25^
	Total	100 (84)	

### *M. massiliense* Clone 1 isolates from the CO-RDP CF-NTM collection

A total of 107 *M. massiliense* isolates from 64 patients were previously analyzed by WGS in a population genomics study by the CO-RDP^9^. Isolates were collected between 2013 and 2018, and patients were associated with CF facilities in 14 US states. Based on phylogenomic analyses, only 11 of 64 (17%) patients with *M. massiliense* had Clone 1 isolates in the previous study^9^. WGS data for a subset of 41 CO-RDP isolates, including Clone1 and non-Clone 1, were included in the current study (Table 1). Clone 1 isolates in the current study include 6 isolates from 6 patients (one isolate per patient) and two isolates per patient from three unique patients. Non-Clone 1 isolates in the current study include 21 isolates from 21 patients (one isolate per patient) and two isolates per patient from four unique patients.

### Genomic isolate cohort

In addition to isolates sequenced for this study, existing WGS data for Clone 1 and non-Clone 1 *M. massiliense* isolates were included in the analysis cohort. These include Clone 1 and non-Clone 1 isolates from 12 previously published studies (Table 1). Clone 1 isolates from known outbreaks include eight isolates from five CF patients at the Papworth Hospital, UK^3^, three isolates from three CF patients treated at a lung transplant and CF center in Seattle, WA^2,4^ and two isolates from an epidemic of soft tissue infections in Brazil from Rio de Janeiro^6,7,22^ and Para^10^. Clone 1 isolates not related to known outbreaks were included from CF patients in two locations in the UK^19,20^, three countries from the global *M. abscessus* population study^8^, and from US CF centers across 14 states^9^. Non-Clone 1 *M. massiliense* isolates with WGS, including the *M. massiliense* type strain CCUG48898^T^^23^, two Asian isolates, and 29 CO-RDP isolates, were added as study controls.

### Genomic variant and phylogenetic analyses

*M. massiliense* genomes (n = 100; Table 1) were compared using a reference based mapping and variant calling approach described previously^26^ with a few modifications. Raw sequence reads were trimmed using Skewer^27^. For publicly available assembled genomes, in silico generated reads were created for mapping. Trimmed and in silico-generated reads were mapped to the complete reference *M. abscessus* genome ATCC19977^T^^28^ using Bowtie2^29^. SNPs relative to the reference genome were called with samtools mpileup v1.5 and bcftools v1.3.1^30^. Base calls were filtered based on mapping quality ≥ 20, a minimum read depth of 4× and a minimum of 75% of reads supporting the base. Only genomic positions with complete genotype data for all isolates in the study were included in downstream analyses. A multifasta sequence alignment was created from concatenated base calls and phylogenetic trees were generated using the neighbor joining method and 100 bootstrap replicates with Seaview^31^. Phylogenetic tree visualizations were created with ggtree^32^. Pairwise distances of core genome SNPs were estimated between isolates using MEGA^33^.

AMR SNPs in the 16S rRNA gene for aminoglycoside (amikacin) resistance^34^ and the 23S rRNA gene for macrolide (clarithromycin) resistance^35^ were extracted from the genome-wide genotype matrix based on genomic coordinates in the ATCC19977^T^ genome The 16S rRNA A1408G mutation corresponds to genomic position 1,463,772, and 23S mutations 2058/2059 correspond to genomic positions 1,466,477 and 1,466,478. *M. massiliense* AMR mutation genotypes were compared using Fisher’s exact tests in R 3.4.3^36^.

### Pan genome analyses

Trimmed reads were assembled into draft genomes using Unicycler^37^, and genes were predicted and annotated using Prokka^38^. For publicly available assembled genomes, genes were also analyzed with the Prokka pipeline to enable consistent gene prediction across all genomes in the dataset. To ensure consistent quality of genome assemblies included in the pan genome analyses, assemblies were compared for overall genome size and number of predicted genes. Genomes with more than two standard deviations from the mean for at least one variable were declared outliers and removed from the analysis. Genome sizes were compared with a t-test in R 3.4.3^36^. Pan genome analyses were performed with Roary^39^.

## Results

### Prevalence of the *M. massiliense* Clone 1 in a mycobacteriology reference laboratory

Between November 2014 and August 2017, a total of 147 patients with isolates of *M. massiliense* were identified at the UTHSCT. Based on the presence of the two characteristic *rpoB* mutations and the *secA1* mutation previously described^2^, a total of 114 isolates obtained from 57 patients (22 males, 38.6% and 35 females, 61.4%) belonged to Clone 1. A total of 132 isolates from 90 patients did not have the characteristic mutations in *rpoB* and *secA1* and were classified as non-Clone 1.

Clone 1 isolates were from respiratory sites (50, 87.7%), wounds (6, 10.5%), and blood (1, 1.8%) from 11 states. Of the 50 respiratory isolates, 10 were bronchoscopy samples, three were tracheal aspirates, and one was a lung biopsy. The remaining 36 samples were sputum cultures. Eighteen of the 50 patients submitting respiratory samples (36%) were identified as having CF. The mean age of the 18 confirmed CF patients was 22.5 years (range 10–44 years) while the mean age of the 17 non-confirmed CF (no diagnostic information was available) patients under age 50 was 31.3 years (range 6–49 years). However, based on their age (under age 50), the 17 patients were considered to have a presumptive (non-confirmed) CF diagnosis. The mean age of all respiratory patients over age 50 was 67.6 years (range 52–89 years).

### Genomic relationships of *M. massiliense* isolates

**Figure 1:**
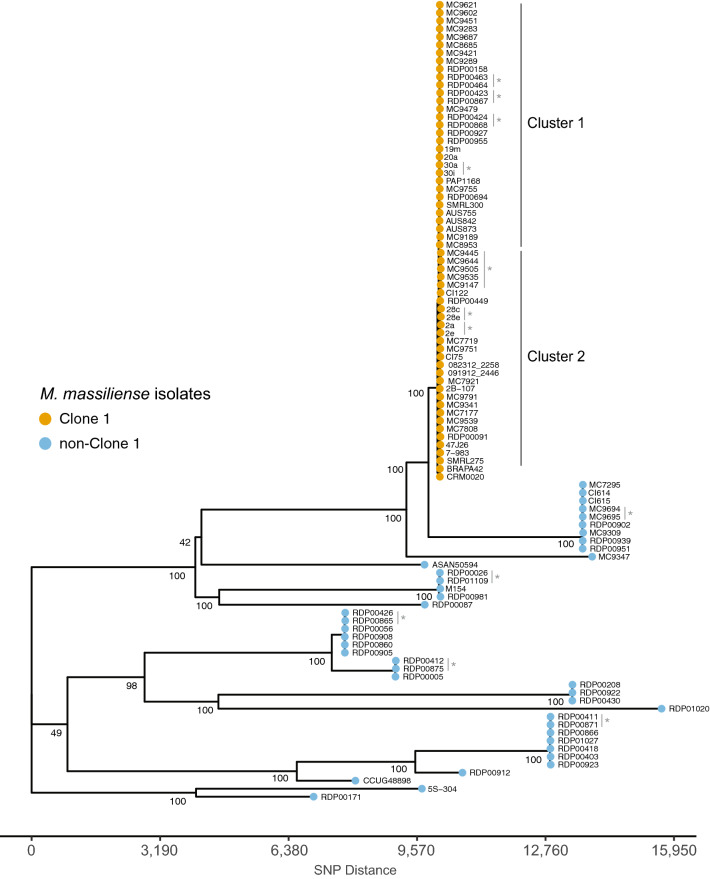
Phylogenomic analysis of *M. massiliense* isolates in the study (n = 100). Genome-wide SNP data at 63,841 positions was analyzed using the neighbor-joining algorithm. Bootstrap support values for the basal nodes are shown. The *M. massiliense* type strain CCUG48898^T^ is included among non-Clone 1 isolates. Longitudinal isolates are shown with grey bars and asterisks. Clusters within the Clone 1 clade are labeled on the right.

Phylogenomic analyses of all 100 isolates in the study cohort were performed to confirm previously observed relationships of *M. massiliense*. A phylogenomic tree, derived from 63,841 genome-wide SNP positions, showed a tightly clustered clade of Clone 1 isolates along with several other clades of genetically diverse *M. massiliense* (Fig. 1). Longitudinal isolates clustered with each other suggesting clonal infections. The placement of the known respiratory outbreak isolates and Brazilian epidemic extrapulmonary strains within the Clone 1 group, and the locations of the type strain, CCUG48898^T^ along with previously described isolates (i.e. ASAN50594 and M154) as more distantly related from Clone 1 suggest a robust phylogeny consistent with previous studies^2,3,26,40^.

### Population structure of *M. massiliense* Clone 1 isolates

**Figure 2:**
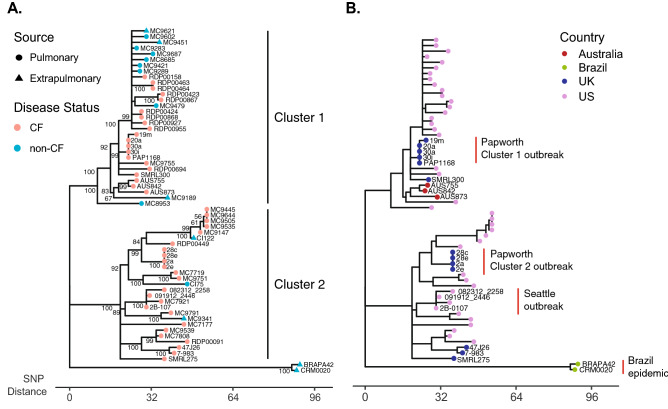
Phylogenetic relationships of Clone 1 isolates. (**A**) Phylogenetic reconstruction of Clone 1 isolates (n = 58) and two Brazilian epidemic isolates (n = 2) was generated using core genome SNP data and the neighbor-joining method. Bootstrap support values (> 50%) after 100 replicate searches are shown. Isolates are labeled by sample source and patient disease status. (**B**) The same phylogeny is labeled by isolate country of origin and previously studied outbreaks are labeled with red bars.

Of the 63,841 variant genomic positions in the full dataset, 564 positions vary among Clone 1 isolates including the two Brazilian isolates. When excluding the Brazilian isolates, only 472 positions vary among Clone 1 isolates (n = 58). A phylogenetic reconstruction of the Clone 1 subgroup shows two distinct lineages, including Cluster 1 and Cluster 2 observed previously in a single-center study from the Papworth Hospital in the UK^3^ and a subsequent study of the Seattle outbreak^2^ (Fig. 2A). Isolates from known outbreaks are in the expected clusters including Papworth isolates (19m, 20a, 30a, 30m) in Cluster 1^3^, and Papworth isolates (2a, 2e, 28c, 28e) and Seattle outbreak isolates (2B-0107, 082312_2258, 091912_2446) in Cluster 2^2^ (Fig. 2B). Both clusters include sporadic isolates sequenced in the current study including CF, non-CF and extrapulmonary isolates. Geographically, Cluster 1 includes isolates from the UK, Australia and six US states. Cluster 2 includes isolates from three locations in the UK and eight US states. Temporally, isolates in Cluster 1 were collected between 2001 and 2017, and isolates in cluster 2 were collected between 2005 and 2017. The Brazilian soft tissue isolates group outside of the two Clusters by more than 100 SNPs, and no similar isolates were identified in this study.

### Antimicrobial resistance mutations in *M. massiliense* and Clone 1 isolates

To assess the prevalence of drug resistance mutations among subgroups of *M. massiliense* isolates, we evaluated known mutations in the 16S rRNA conferring amikacin resistance and the 23S rRNA for clarithromycin resistance in our genomic isolate cohort. Using one isolate per patient, we first compared Clone 1 (n = 46) vs. non-Clone 1 isolates (n = 38). While we observed higher proportions of AMR mutations in the 16S and 23S rRNAs in Clone 1 compared to non-Clone 1 isolates, they were not statistically significant; amikacin resistance mutation (p = 0.21) and clarithromycin resistance mutations (p = 0.06) (Supplementary Figure 1A).

Next, we compared the prevalence of drug resistance mutations within the Clone 1 subgroup, between isolates from patients associated with pulmonary outbreaks (n = 7) and sporadic isolates (n = 39) and found that drug resistance mutations were significantly higher among outbreak isolates for both amikacin (p = 0.0016) and clarithromycin (p < 0.0001) compared to non-outbreak strains (Supplementary Fig. 1B). Brazilian epidemic isolates do not have these AMR mutations.

### Pan genome of *M. massiliense* Clone 1 isolates

**Figure 3:**
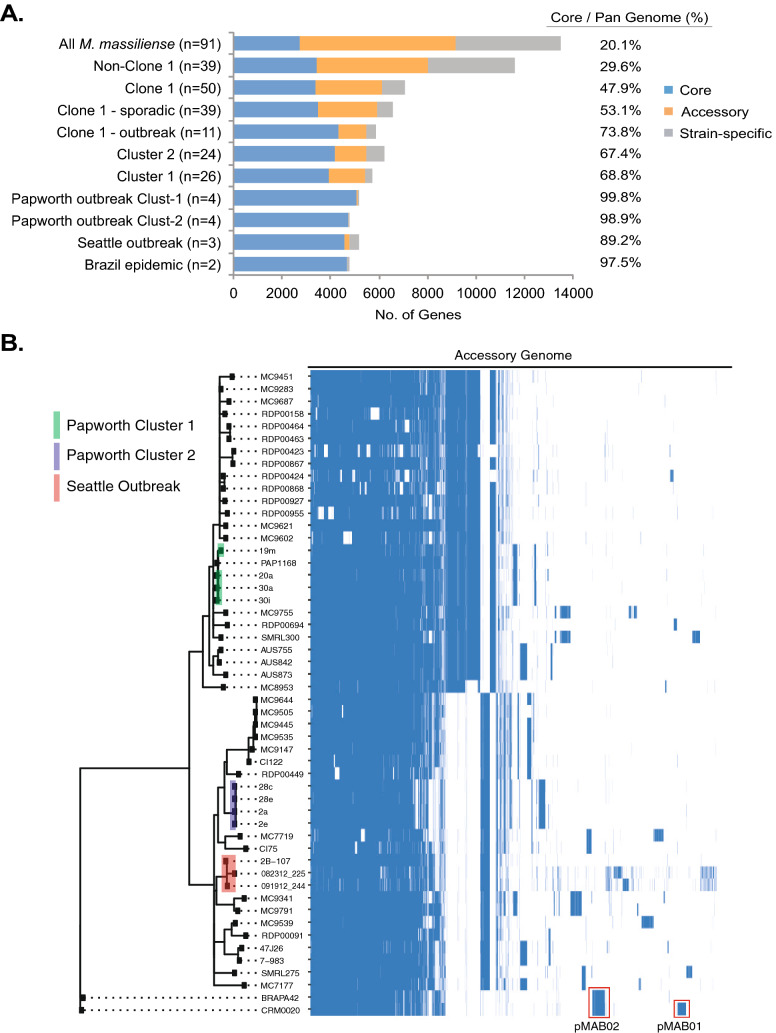
Pan genome of *M. massiliense*. Pan genome analyses were performed for *M. massiliense* isolates that passed genome assembly quality filters (see “Methods”; n = 91). (**A**) Pan genome results for all isolates in the dataset and various isolate subgroups are shown as numbers of core genes, accessory genes and strain-specific genes. Percent of core genes for each group is shown (core genes/ total genes in pan genome). (**B**) Visualization of the accessory genome for Clone 1 *M. massiliense* isolates. Accessory genes for Clone 1 isolates (n = 50) and Brazilian epidemic isolates (n = 2) are shown as a presence or absence heatmap (blue means presence of a gene). Samples are ordered based on the core genome phylogenetic tree (left side). Known outbreak isolate clades are color coded for reference, and plasmids are indicated with red boxes.

To explore genomic variation beyond the core genome, we performed pan genome comparisons for all *M. massiliense* that met our genome assembly quality criteria (n = 91). Among the entire genomic dataset, *M. massiliense* genomes were, on average, 4.98 Mb in size (range 4.7–5.4 Mb) similar to previous studies^10,40^, with an average of 5011 predicted genes per genome (range 4609–5517). There was no significant difference in average genome size between Clone 1 and non-Clone 1 isolates (t-test, p = 0.68). Using predicted protein coding genes for each genome, we identified gene presence/absence variation among the entire dataset and subsets of isolate genomes (Fig. 3A). Among all isolates (n = 91), the pan genome included 13,460 unique genes of which only 2715 genes made up the core genome shared by all isolates, consistent with previous findings in *M. massiliense*^40^. Among the genetically diverse non-Clone 1 isolates (n = 39), the pan genome included 11,583 unique genes with a core genome of 3430 genes (30% core genes). In contrast, the genetically similar Clone 1 isolates (n = 50) had a smaller pan genome of 7041 unique genes and a core genome of 3375 genes (48% core genes).

Within Clone 1, isolates in Cluster 1 (n = 26) and Cluster 2 (n = 24) showed pan genomes of 6193 and 5707 genes, with core genomes of 3929 and 4175 genes (69% and 67%), respectively. Clone 1 sporadic isolates (n = 39) had a smaller core genome (53.1%) compared to Clone 1 outbreak isolates (n = 11; 73.8%). Finally, isolate subsets from known pulmonary outbreaks showed highly similar genomes with core genes ranging from 89% (Seattle) to 99% (Papworth Cluster 2). The two Brazilian epidemic isolates had highly similar genomes (97% core genes) and differed only by the presence or absence of the pMAB01 plasmid.

A heatmap of the accessory genome (Fig. 3B) for Clone 1 isolates (n = 50) and Brazilian epidemic isolates (n = 2) revealed the gene presence/absence variation among highly similar isolates by the core genome. The heatmap shows clusters of genes that are specific to Cluster 1 or Cluster 2, in addition to strain-specific genes in various isolates within the clusters. The analysis also identified two plasmids among the Brazilian epidemic isolates, pMAB01^11^ and pMAB02^10^, which were absent in the genomes of all other isolates in our study in contrast to a previous study that identified pMAB02 in a small proportion of CF-related pulmonary isolates^10^.

### Outbreak versus sporadic Clone 1 isolates

**Figure 4:**
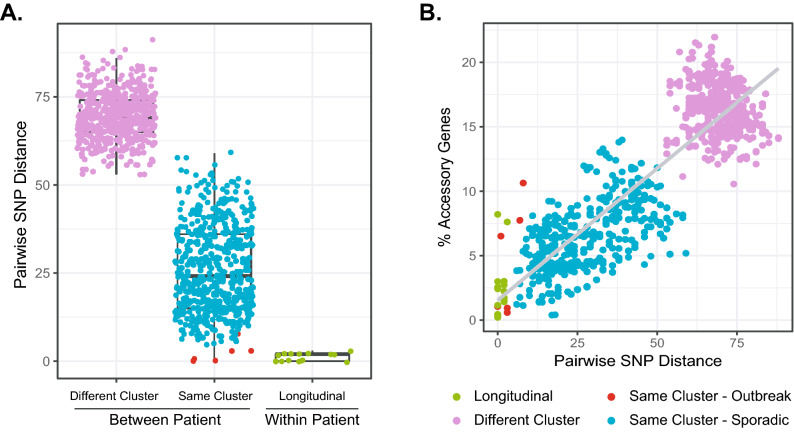
Pairwise genomic variation among Clone 1 *M. massiliense* isolates. Pairwise SNP distances were calculated for all Clone 1 isolates in the study (n = 58). (**A**) Isolate pairs are categorized as between-patient or within-patient. Between patient isolates in the same cluster (Cluster 1 or Cluster 2) are further categorized as belonging to known outbreaks (red) or as sporadic, unrelated isolates (blue). (**B**) The relationship between accessory gene content for Clone 1 isolate pairs, measured as % accessory genes for each isolate pair (# accessory genes/# of genes in pan genome) and core genome similarity (shown as pairwise SNP distance) is shown as a scatter plot.

We evaluated thresholds of genomic variation between patient isolates associated with previous outbreaks (one isolate per patient including Brazilian strains; n = 10) versus those classified as sporadic Clone 1 isolates (one isolate per patient; n = 31). As a reference point, pairwise SNP distances between longitudinal same-patient isolate pairs (n = 8 patients) were also calculated (Fig. 4A). Pairwise SNP distances ranged from 0 to 3 SNPs for same-patient isolates, 0–8 SNPs between patients in known outbreaks (Papworth, Seattle and Brazil), 6–59 SNPs between sporadic isolate pairs in the same cluster (Cluster 1 or Cluster 2 as in Fig. 2), and 51–91 SNPs between patient isolates in different clusters (Fig. 4A).

To understand the relationship between the core genome (measured by SNPs) and accessory genome variation (measured by gene presence/absence) among highly similar Clone 1 isolates, we compared pairwise SNP distances (Fig. 4A) and % accessory genome variation for all isolate pairs (Fig. 4B). While there is an overall positive relationship between SNP distance and % accessory genome in the population, there is substantial variation in the accessory genome among isolate pairs that are highly similar in the core genome. For example, longitudinal, same-patient isolate pairs had an average of 2.5% accessory genome variation (range 0.2–8.2%; time span range: 2–246 days). Papworth pulmonary outbreak isolates were highly similar and showed 0.6–1.1% accessory genome variation between patients, while the Seattle outbreak isolates showed 6.5–10.6% accessory genome variation between patients. Sporadic isolate pairs that were highly similar at the core genome level (less than 10 SNPs) included CF/CF and non-CF/CF pairs, and showed accessory genome variation of 1.1–6.2% which was well within the range of longitudinal and outbreak isolate thresholds in our study.

## Discussion

This study demonstrates that the *M. massiliense* dominant Clone 1 is not limited to the respiratory tract of patients with CF, but is also found in sporadic wound infections, blood stream infections, and the respiratory tract of primarily older women. Clone 1 was identified in multiple states across the US, in two clinical mycobacteriology laboratories, and in a nationwide CF-NTM biorepository^9^. At the UTHSCT, the prevalence of Clone 1 among all isolates of *M. massiliense* was surprisingly high compared to the CO-RDP (39% vs. 17%). The difference in prevalence between patient isolates sent to the UTHSCT and CF isolates sent to the CO-RDP may reflect differences in disease states (non-CF vs. CF), geographic distribution of isolates, suspicion of outbreaks, or other sampling biases.

Pan genome analyses showed that *M. massiliense* has broad diversity in gene content similar to previous studies of *M. abscessus*^40^. We observed genes that vary within the Clone 1 lineages, Clusters 1 and 2 (Fig. 3B), illustrating that isolates which appear ‘clonal’ by MLST or core genome analysis also have underlying genomic variation through gene loss and gain^26,41^. This is consistent with previous observations among highly similar *M. abscessus* clusters^42^. While accessory genome variation broadly tracks with core genome (SNP) variation (Fig. 4B), it can vary widely among isolates with highly similar core genomes. For instance, the Papworth outbreak clusters showed very little accessory genome variation (< 2%) while the Seattle outbreak isolates showed more diversity between the index case and other cases (~ 10%). The contrast in genomic variation between outbreak scenarios may reflect in vivo diversification of sublineages in the index case that were subsequently transmitted to other patients. Clonal sublineages within patients have been observed in long-term *Pseudomonas aeruginosa* infections^43,44^, and one longitudinal patient in our sample set showed similar accessory genome variation (Fig. 4B). Our results suggest that the pan genome offers additional and useful information for deconstructing relationships between highly similar isolate clusters.

Through this study, we identified highly similar sporadic isolate pairs (< 10 SNPs between patients) including those between CF and non-CF patients and between pulmonary and extrapulmonary isolates. Many of these pairs do not have obvious connections or opportunities for cross infection as patients were from different states and/or separated by months or years of time. This corresponds to findings from the CO-RDP where CF patients from widely separated geographic regions shared highly similar *M. abscessus* isolates^9,21^. Genomic studies within single CF centers have largely corroborated these findings through lack of epidemiological evidence for healthcare associated transmission among highly similar isolate clusters^19,42,45^. This underscores the uncertainty of using genetic similarities alone to presume person-to-person transmission. Epidemiological investigations including environmental sampling are needed in parallel to identify inoculum sources and rule out patient cross transmission.

The rarity of acquired AMR mutations among sporadic US Clone 1 isolates in our study suggests that they could be useful indicators of cross transmission between patients. One interesting observation in our study is a CF patient from the Seattle, WA area that presented to the University of Washington CF Center for the first time in 2012 with an amikacin and clarithromycin resistant isolate. The 2012 isolate clustered with the Seattle CF outbreak cluster by core genome similarity (Fig. 2; MC7921) but the patient had no known contact with the Seattle outbreak cases diagnosed in 2004 and 2005^4^. This suggests the outbreak clone may still exist, perhaps in the local environment, though no additional cases have been identified. The high proportion of AMR mutations observed among outbreak isolates compared to sporadic isolates suggests that they may be fitness factors promoting long term infection and opportunities for cross-infection between patients^8^. However, additional outbreak isolates would be needed to validate this hypothesis.

While many insights have been gained from our analysis of sporadic US *M. massiliense* isolates, there are limitations to the study. First, we have a low sample size of known outbreak isolates to compare with sporadic isolates. Second, we have incomplete knowledge of residential history and initial NTM acquisition for patients in the study, and therefore cannot fully evaluate the potential for shared exposure or cross infection. That the dominant clone is found in non-CF and CF settings across the US suggests that it is not specific to CF centers and may be widespread in the environment or man-made systems. Complementary environmental studies are needed to identify precise locations of *M. massiliense* and other rapidly-growing NTM in the environment.

## Supplementary Information

Supplementary Figure 1.

## Data Availability

WGS data generated for this study are available at National Center for Biotechnology Information (NCBI) under BioProject PRJNA691364.
