# Supplementary material for: Genomic characterization of sporadic isolates of the dominant clone of Mycobacterium abscessus subspecies massiliense
Source: Sci Rep. 2021 Jul 28;11:15336. doi: 10.1038/s41598-021-94789-y (PMC8319421; doi:10.1038/s41598-021-94789-y)
Supplement: Supplementary file 1 — Supplementary Figure 1. [file 41598_2021_94789_MOESM1_ESM.pdf]

SUPPLEMENTARY INFORMATION FOR

Genomic Characterization of Sporadic Isolates of the Dominant Clone of *Mycobacterium abscessus* subspecies *massiliense*

Rebecca M. Davidson[1], Jeanne B. Benoit[1], Sara M. Kammlade[1], Nabeeh A. Hasan[1], L. Elaine Epperson[1], Terry Smith[2], Sruthi Vasireddy[2], Barbara A. Brown-Elliott[2], Jerry A. Nick[3], Kenneth N. Olivier[4], Adrian M. Zelazny[5], Charles L. Daley[3], Michael Strong[1], Richard J. Wallace Jr.[2]

Running Title: Dominant Clone of *Mycobacterium massiliense*

1. Center for Genes, Environment and Health, National Jewish Health, Denver, CO
2. Department of Microbiology, University of Texas Health Science Center at Tyler, Tyler, TX
3. Department of Medicine, National Jewish Health, Denver, CO
4. Pulmonary Branch, National Heart, Lung, and Blood Institute, National Institutes of Health, Bethesda, MD
5. Department of Laboratory Medicine, Clinical Center, National Institutes of Health, Bethesda, MD

Corresponding Author:
Rebecca M. Davidson, PhD
Center for Genes, Environment and Health,
National Jewish Health,
1400 Jackson St., Denver CO 80206.
303-270-2592 (office)
DavidsonR@NJHealth.org

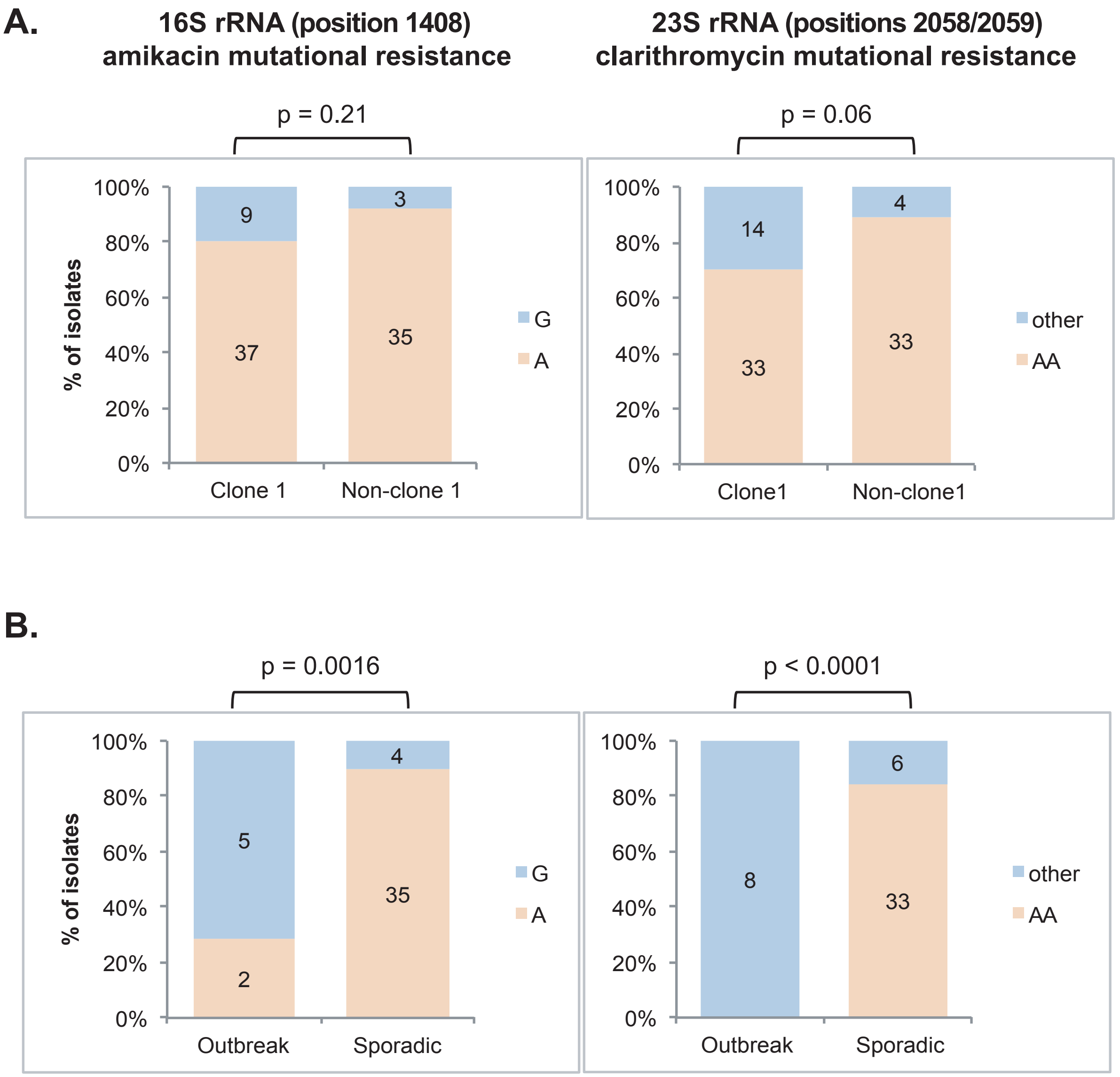

Supplemental Figure 1. Proportions of known antimicrobial resistance (AMR) mutations in *M. massiliense* isolates. The 16S rRNA position 1408 for amikacin mutational resistance and 23S rRNA positions 2058 and 2059 for clarithromycin mutational resistance were analyzed in *M. massiliense* genomes using one isolate per patient. Genotypes in orange are the wild type, and genotypes in blue are the AMR genotype. Percent of isolates are shown on y-axes. **A.** *M. massiliense* isolates are classified as Clone 1 vs. Non-Clone 1. **B.** Clone 1 isolates are classified as Outbreak vs. Sporadic.
